# Effect of Silicon on the Biology and Reproductive Fitness of *Tetranychus macfarlanei* Baker and Pritchard (Acari: Tetranychidae) on the Country Bean (*Lablab purpureus* L.)

**DOI:** 10.3390/plants14121765

**Published:** 2025-06-09

**Authors:** Md. Nasimul Hassan, Faysal Ahmed, Farhana Akter Tonni, Mst. Masuma Momtaj Meem, Quazi Forhad Quadir, Tetsuo Gotoh, Mohammad Shaef Ullah

**Affiliations:** 1 Laboratory of Applied Entomology and Acarology, Department of Entomology, Bangladesh Agricultural University, Mymensingh 2202, Bangladesh; nasimulhassan16@gmail.com (M.N.H.); faysalahmed1936@gmail.com (F.A.); tonni.1902261@bau.edu.bd (F.A.T.); meem.1902092@student.bau.edu.bd (M.M.M.M.); 2 Laboratory of Plant Nutrition and Environmental Chemistry, Department of Agricultural Chemistry, Bangladesh Agricultural University, Mymensingh 2202, Bangladesh; qfq@bau.edu.bd; 3 Faculty of Agriculture, Ibaraki University, Ami, Ibaraki 300-0393, Japan

**Keywords:** defense mechanism, silicon, reproduction, life table, spider mite management

## Abstract

The red spider mite, *Tetranychus macfarlanei*, is a significant pest of various crops, and silicon (Si), a beneficial micronutrient, serves as a physical defense against herbivores when accumulated in plant tissues. This study examined the effects of silicon on the biology of *T. macfarlanei* on *Lablab purpureus* plants treated with 0 ppm (control), 28, and 56 ppm silicon concentrations. The results showed that silicon treatments notably affected mite development. At the highest concentration of 56 ppm Si, females exhibited the longest immature period, shortest lifespan, and shortest oviposition period. Egg production per female was highest at the 0 ppm Si level (94.62) and lowest at the 56 ppm Si concentration (42.29). Life table parameters, including the intrinsic rate of increase (*r*), net reproductive rate (*R*_0_), finite rate of increase (λ), and gross reproductive rate (*GRR*), declined progressively with increasing silicon concentrations. Compared to the control (0 ppm Si), the highest silicon level resulted in reductions of approximately 24% in *r*, 55% in *R*_0_, 4% in *λ*, and 27% in *GRR*, indicating a substantial negative impact of silicon on the reproductive potential of *T. macfarlanei*. These findings suggest that higher silicon levels effectively suppress *T. macfarlanei* populations and may be useful in integrated mite management strategies.

## 1. Introduction

The red spider mite, *Tetranychus macfarlanei* Baker and Pritchard (Acari: Tetranychidae), is an economically significant pest responsible for substantial yield losses in various agricultural crops, particularly within the Malvaceae, Fabaceae, Cucurbitaceae, Convolvulaceae, and Solanaceae families [1,2,3]. It is increasingly recognized as a notorious polyphagous pest with widespread distribution across Tropical, Oriental, and Palearctic regions, including Bangladesh, India, China, Malaysia, and other countries [1,2].

This mite commonly infests a wide range of crops including vegetables, fruits, tea, cotton, and ornamentals, feeding primarily on the undersides of leaves. The feeding activity reduces chlorophyll and moisture content, causing visible symptoms such as yellow stippling, bronzing, leaf bleaching, and, ultimately, significant crop yield reduction [4,5,6].

While synthetic acaricides are traditionally used for mite control, their prolonged use raises serious concerns, including pesticide resistance, non-target effects on beneficial organisms, and environmental pollution [7,8]. These issues have prompted the need for sustainable alternatives in mite management.

Silicon (Si) is widely present in the Earth’s crust, and has garnered attention for its role in enhancing plant resistance to biotic and abiotic stresses. While it is not considered an essential nutrient, Si contributes to plant structural integrity and defense mechanisms. Its deposition in plant tissues can deter herbivory by increasing leaf toughness and abrasiveness [9]. Furthermore, Si supplementation has been shown to induce the production of defense-related enzymes and secondary metabolites, thereby enhancing plant resistance to pests and diseases [6].

Recent studies have demonstrated the efficacy of Si in mitigating infestations of the two-spotted spider mite (*Tetranychus urticae*). For instance, foliar applications of silicon compounds on strawberry plants resulted in significant reductions in mite populations and enhanced activity of defense-related enzymes [6]. Similarly, Si treatments have been associated with alterations in plant volatile emissions, leading to increased attraction of natural enemies and improved biological control of pests [10]. Indeed, field studies have shown that foliar application of silicon-based fertilizers reduced populations of various mite species such as *Oligonychus sacchari* on sugarcane, supporting its inclusion in integrated pest management (IPM) programs [11]. Despite these promising findings, research on the impact of Si on *T. macfarlanei*, particularly on the country bean (*Lablab purpureus*), remains limited. Given the pest’s economic significance and the need for sustainable management strategies, it is imperative to explore alternative control measures.

While the role of silicon in enhancing plant resistance against various pests, including *T. urticae*, has been documented, there is a paucity of information regarding its effects on *T. macfarlanei*. Specifically, studies investigating the influence of Si on the biology and reproductive fitness of *T. macfarlanei* on country bean are lacking. Bridging this gap is essential for formulating integrated pest management strategies that are both effective and environmentally sustainable. This study aims to investigate the impact of different silicon concentrations on the biology and reproductive fitness of *T. macfarlanei* on country bean. The findings are expected to provide insights into the potential of Si as a component of integrated mite management programs.

## 2. Results

### 2.1. Silicon Accumulation in Host Plant Leaves

Application of different concentrations of silicon significantly influenced its accumulation in bean leaves used for rearing *Tetranychus macfarlanei* (Table 1). The highest silicon concentration was observed in leaves from the high silicon treatment group (398.60 ppm), followed by the low silicon treatment (161.20 ppm), and the control (116.60 ppm). This confirmed effective absorption and accumulation of silicon in the plant tissue, establishing the foundation for its potential effects on mite biology.

### 2.2. Immature Development

The developmental duration of *T. macfarlanei* varied significantly with different silicon treatments (Table 2). High silicon concentration (56 ppm) resulted in a significantly shorter egg period in females (5.94 ± 0.11 days) compared to the control group (6.53 ± 0.14 days), while the low silicon (28 ppm) group (6.30 ± 0.17 days) showed an intermediate value that was not significantly different from either. However, the larval stage lasted longest in the high Si treatment for both sexes.

The total immature development duration (egg to adult) of male *T. macfarlanei* was significantly extended under the high silicon treatment (15.19 days) compared to the low (14.42 days) and control (13.88 days) treatments (*p* < 0.05). Although female developmental duration also exhibited a numerical increase under high silicon conditions (15.00 days) relative to the low (14.77 days) and control (14.47 days) groups, these differences were not statistically significant.

### 2.3. Adult Longevity and Reproduction

Silicon treatments significantly influenced adult longevity and reproductive output in female *T. macfarlanei* (Table 3). The total pre-oviposition period (TPOP) was significantly longer under high Si treatment (16.92 days) compared to low Si (16.41 days) and control (16.03 days). However, oviposition period and total female longevity were maximized under low silicon treatment (19.07 and 37.77 days, respectively), suggesting a hormetic effect at low doses.

Egg production was significantly reduced in the high silicon group (42.29 eggs per female) compared to low (95.53) and control (94.62), indicating that elevated silicon levels adversely affect mite fecundity.

### 2.4. Age-Stage, Two-Sex Life Table Parameters

**Figure 1:**
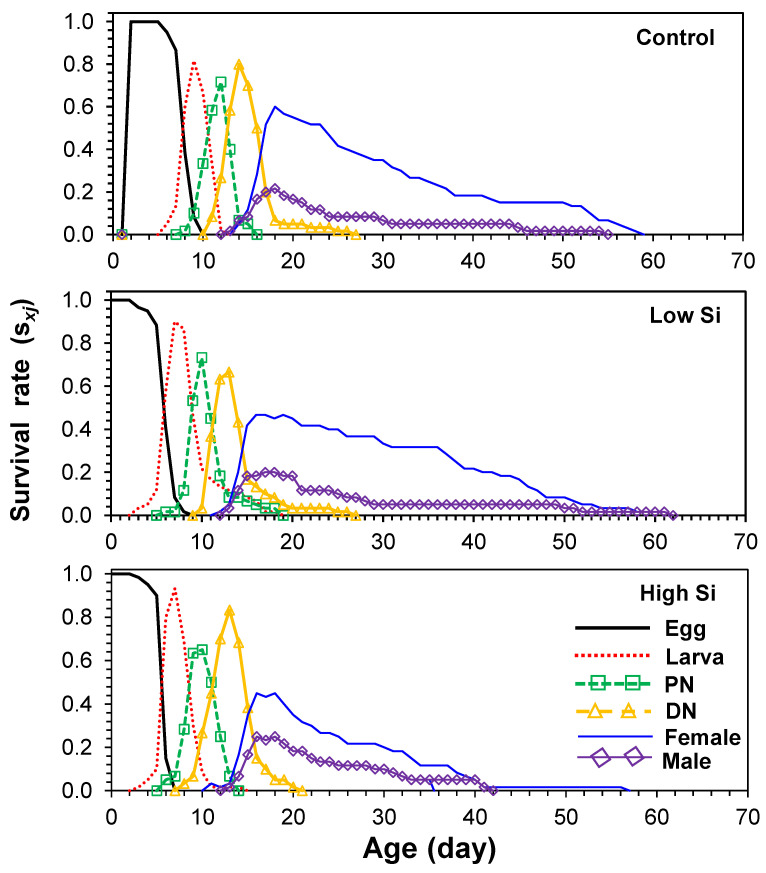
Age-stage-specific survival rates (*s_xj_*) of *Tetranychus macfarlanei* reared on different treatments of Silicon on bean plants at 25 °C and 65 ± 5% relative humidity.

**Figure 2:**
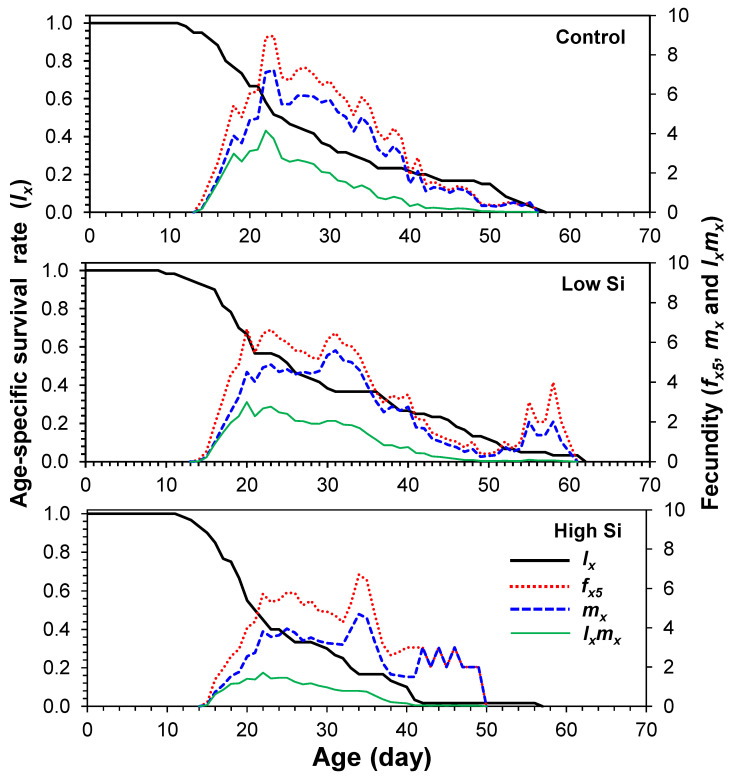
Age-specific survivability (*l_x_*), age-stage-specific fecundity (*f_x_*), age-specific fecundity (*m_x_*), and age-specific maternity (*l_x_m_x_*) of *Tetranychus macfarlanei* reared on different treatments of Silicon on bean plants at 25 °C and 65 ± 5% relative humidity.

Age-stage-specific survival curves (*s_xj_*) showed stage overlap among cohorts, with survivorship declining faster in the high silicon treatment (Figure 1). Age-specific fecundity (*m_x_*) and maternity (*l_x_m_x_*) curves (Figure 2) indicated that oviposition began on days 13–14 and peaked at different times across treatments: day 22 (control), day 32 (low), and day 35 (high).

### 2.5. Population Parameters

Population growth metrics showed significant reductions in intrinsic rate of increase (*r*), net reproductive rate (*R*_0_), and finite rate of increase (*λ*) in the high silicon group (Table 4). The control group exhibited the highest *r* (0.1679 day^−1^), *R*_0_ (53.62), and *λ* (1.1829), while the high silicon group showed the lowest *r* (0.1281 day^−1^) and *R*_0_ (23.97), indicating strong suppressive effects of silicon on mite population growth. The mean generation time (*t*) was longest in the low silicon treatment (25.10 days), followed by high silicon (24.81 days), and shortest in the control (23.71 days). *GRR* was highest in the control group (126.30), slightly lower in the low silicon group (116.17), and lowest in the high silicon group (92.66).

### 2.6. Life Expectancy and Reproductive Value

**Figure 3:**
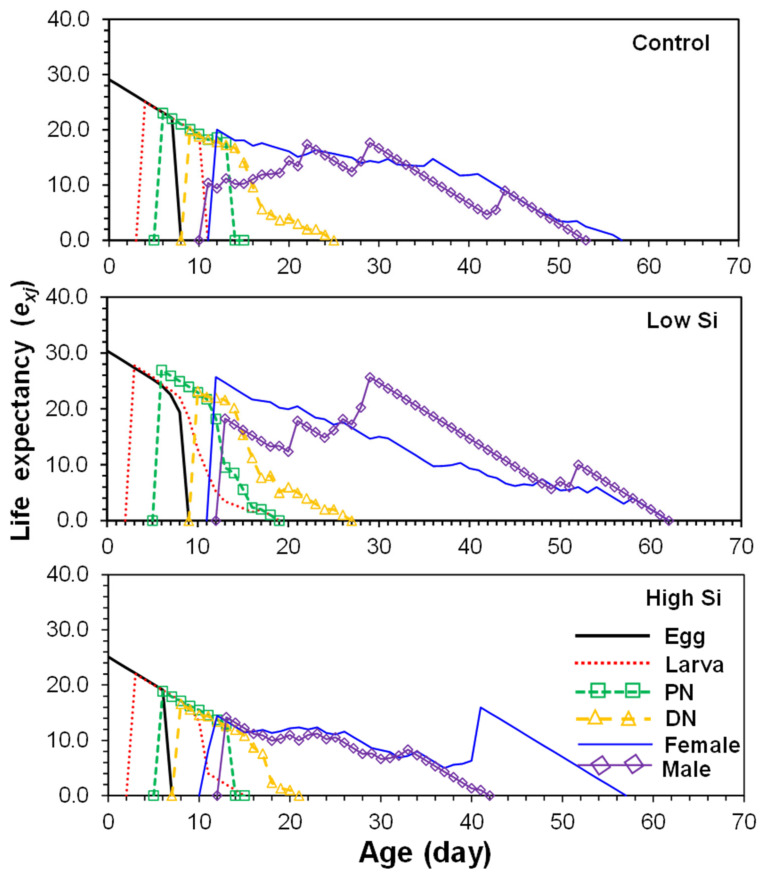
Age-stage life expectancy (*e*_xj_) of *Tetranychus macfarlanei* reared on different treatments of Silicon on bean plants at 25 °C and 65 ± 5% relative humidity.

Life expectancy (*e_xj_*) of newly hatched eggs was highest in the low silicon group (30.2 days), followed by control (29.5 days) and high silicon (25.0 days) (Figure 3). Peak life expectancy of adult females ranged between 11 and 12 days across treatments.

**Figure 4:**
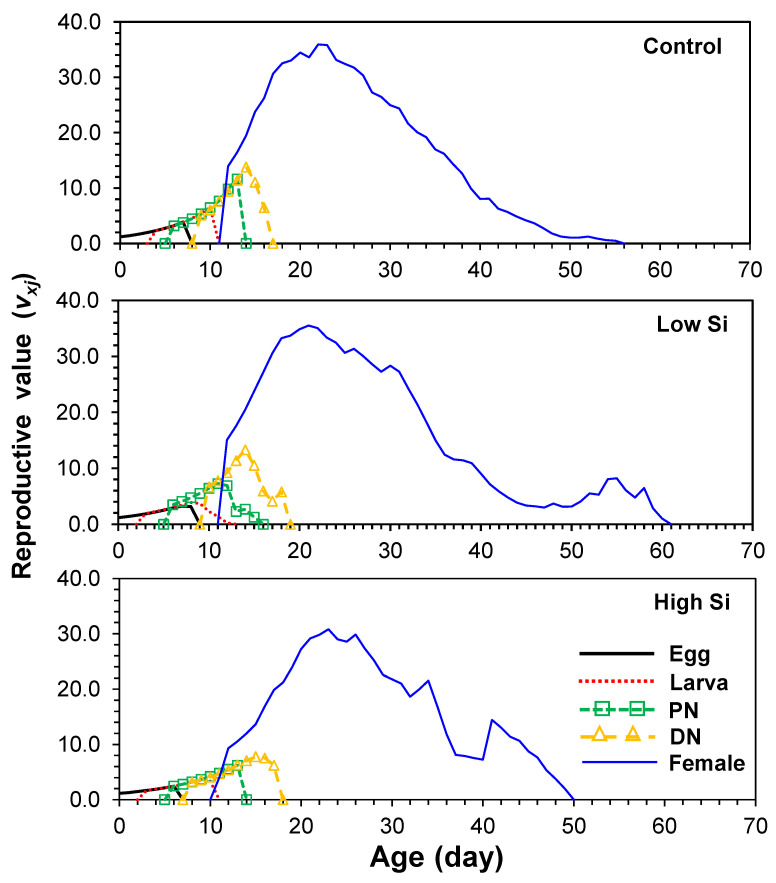
Age-stage fecundity (*v_x_*_j_) of *Tetranychus macfarlanei* reared on different treatments of Silicon on bean plants at 25 °C and 65 ± 5% relative humidity.

Reproductive value (*v_xj_*) of newborns was lowest in high silicon (1.1) compared to 1.2 in both control and low groups. The peak reproductive value for adult females was 23 in both control and high treatments and 22 in the low silicon group (Figure 4).

## 3. Discussion

This study demonstrates that *Tetranychus macfarlanei* exhibits significant biological and demographic responses to silicon application, particularly at higher concentrations. Silicon at 56 ppm (high Si concentration) markedly reduced longevity, fecundity, oviposition period, and key life table parameters, underscoring its role as a defensive agent against herbivorous arthropods on country bean (*Lablab purpureus*). These findings indicate that elevated silicon levels can impede developmental progression in *T. macfarlanei*, particularly in males, potentially contributing to delayed population growth under silicon-enriched conditions. These results align with previous findings on *Tetranychus urticae*, where silicon supplementation suppressed oviposition and population growth while mitigating plant damage and enhancing physiological resilience [11]. Additionally, the enhanced attraction of predatory mites to herbivore-induced plant volatiles (HIPVs) from Si-treated plants further supports silicon’s dual role in plant defense through both direct and indirect mechanisms.

The findings corroborate previous research conducted on *T. urticae*, a closely related species. For example, a significant decline in fecundity and population growth of *T. urticae* was found with Si sprayed on leaves of papaya [12] and cucumber [13], respectively. Similarly, it was demonstrated that foliar application of silicon in strawberries significantly reduced *T. urticae* population density while enhancing defense-related enzyme activity (peroxidase, catalase, and polyphenol oxidase), indicating silicon’s role as a physiological defense activator [6].

Our study further strengthens these findings by showing that the gross reproductive rate (*GRR*) and net reproductive rate (*R*_0_) were markedly lower in mites reared on high-Si-treated plants. These demographic shifts can be attributed to both direct and indirect impacts of silicon on host plant quality. It was reported that silicon sprayed on leaves induced stress responses in strawberry leaves, prolonging immature developmental stages and disrupting oviposition timing in *T. urticae* [14].

One potential mechanism behind these effects is the structural reinforcement of plant tissues. As noted, silicon deposition strengthens the cell wall and cuticle layers, forming silica–cuticle double layers and phytoliths, making feeding physically difficult for herbivores [15]. This not only causes gut abrasions and mandibular wear in chewing insects but also limits nutrient assimilation. These structural barriers, coupled with increased tissue abrasiveness and reduced palatability, are likely to hinder mite feeding efficiency, delay development, and disrupt reproductive outputs.

Moreover, silicon also acts as a chemical defense enhancer, potentially influencing hormonal pathways like the jasmonic acid (JA) signaling cascade. As proposed, JA-mediated signaling—induced or primed by Si—regulates the synthesis of herbivore-induced plant volatiles (HIPVs), which in turn enhance plant attractiveness to natural enemies like predatory mites and parasitoids [11,16,17]. Such volatile emissions (e.g., E-β-ocimene, D-limonene, methyl salicylate) have been shown to increase upon silicon treatment, effectively acting as indirect defenses against herbivores [16,17].

Interestingly, in our study, the mean generation time (*t*) was prolonged under silicon-treated conditions, particularly in the 28 ppm treatment. This reflects a delayed generational turnover in mite populations, which is a significant ecological disadvantage in pest species, especially when combined with reduced intrinsic rates of increase (*r*). These results are in line with findings in [18], which noted that Si supplementation applied as basal amendments in rice resulted in enhanced callose deposition and amino acid changes that may affect pest metabolism and behavior. Amino acids such as asparagine (Asn) can mimic neurotransmitters or disrupt insect protein synthesis, acting as antimetabolites [15].

Additionally, silicon’s influence extends beyond its direct effects on herbivores. By increasing HIPV emissions, it facilitates the tritrophic interaction—improving the effectiveness of biological control agents like the predatory mite *Phytoseiulus persimilis* Athias-Henriot and the red and blue beetle *Dicranolaius bellulus* (Boisduval), as documented [11,19]. Our findings align with this, suggesting that silicon-rich environments may not only stress herbivores physiologically but also increase their visibility and susceptibility to predators and parasitoids.

Moreover, field-based research on *Oligonychus sacchari* McGregor in sugarcane demonstrated that silicon-based fertilizers significantly lowered mite populations across several cultivars without adverse effects on key natural enemies, suggesting broad-spectrum applicability of silicon in mite management [20]. These parallels highlight the versatility of Si as a pest management tool across different cropping systems and mite species [19]. The reduced fecundity and intrinsic rate of increase (*r*) observed in this study echo the demographic shifts seen in silicon-treated plants in previous reports and point to a suppression mechanism likely linked to both impaired feeding efficiency and altered host plant quality. In the present study conducted at 25 °C, the intrinsic rate of increase (*r*) and net reproductive rate (*R*_0_) of *T. macfarlanei* were significantly reduced by potassium silicate treatment, particularly at 56 ppm concentration. The *r* and *R*_0_ values recorded under Si treatment were notably lower than those observed under untreated conditions in earlier studies at the same temperature. *Tetranychus macfarlanei* reported an *r* of 0.275 day^−1^ and *R*_0_ of 167.4 females/female at 25 °C on untreated *Lablab purpureus* [21], while another study found control values of *r* = 0.150 and *R*_0_ = 29.6 at 25 °C, with reductions occurring under sublethal concentrations of spirotetramat [22]. In contrast, our study showed much lower *r* and *R*_0_ values under high Si treatment, indicating a strong inhibitory effect on the mite’s population growth potential. This suppression can be attributed to Si-induced changes such as increased cell wall rigidity and plant defense responses, which likely impaired feeding efficiency, extended development time, and reduced fecundity of the mites. The consistent decline in demographic parameters under silicon application at 25 °C highlights its potential as a sustainable, non-toxic strategy for managing *T. macfarlanei* on country bean. However, one limitation of our study is the sole use of potassium silicate as the Si source. In the present study, potassium silicate was applied as the silicon source, a formulation known for its solubility and plant-available silicon content. However, it is important to note that the type of silicon compound used can significantly influence its efficacy against pests. While our results showed a clear negative impact on the development and reproduction of *T. macfarlanei*, previous studies on silicon’s effects have often employed different formulations such as sodium silicate, calcium silicate, or even foliar-applied silica nanoparticles, which vary in their mode of uptake and bioavailability to plants.

These differences in silicon forms may account for the variability in pest responses reported in the literature. For instance, some studies that observed weaker impacts on herbivores may have used forms less readily absorbed by plants or with slower systemic movement [9]. Therefore, the effectiveness observed in our study using potassium silicate may be attributed in part to its higher uptake efficiency, contributing to both structural and biochemical resistance in *L. purpureus*. Future investigations should evaluate other forms (e.g., rice husk ash, wollastonite, or foliar-applied oligomeric silicic acid) and their efficacy on both mites and plants across different cropping systems.

## 4. Materials and Methods

### 4.1. Plant Material

The experiment utilized certified seeds of country bean (*Lablab purpureus* L.), specifically the cultivar BARI Sheem 8, obtained from a reliable seed source. The seeds were sown in nine circular earthen pots, each with a diameter of 25 cm, filled with sterilized Pro-Mix potting soil. The pots were arranged in a well-ventilated screen house under ambient light conditions. Standard agronomic practices, including routine irrigation, manual weed removal, and pest monitoring, were performed to ensure optimal plant growth. The experiment continued until the plants reached the leaf maturation stage suitable for mite infestation studies.

### 4.2. Experimental Site and Treatments

The study was conducted through a combination of field cultivation and laboratory experiments at the Laboratory of Applied Entomology and Acarology, Department of Entomology, Bangladesh Agricultural University, Mymensingh, Bangladesh. The experimental setup followed a Completely Randomized Design (CRD) with three replications per treatment, arranged under a factorial scheme.

Three treatment groups were applied:

Control (0 ppm Si)—received only distilled water.

Low Si dose (28 ppm Si)—potassium silicate applied at 28 ppm concentration.

High Si dose (56 ppm Si)—potassium silicate applied at 56 ppm concentration.

### 4.3. Preparation and Application of Silicon Solution

Potassium silicate (K_2_SiO_3_) served as the source of silicon. For the high dose (56 ppm), 1 mL of potassium silicate was dissolved in 4 L of distilled water; for the low dose (28 ppm), 1 mL was dissolved in 8 L of distilled water. Since potassium silicate tends to elevate pH, the solutions were adjusted to a pH range of 5.5–6.0 using 2 mM hydrochloric acid (HCl). The prepared solutions were thoroughly mixed and applied as soil drenches to each pot at fortnightly interval. Each silicon (Si) dose was applied fortnightly as a ground drench to the base of the plants for ten consecutive weeks, continuing until the leaves matured. Silicon was applied as a ground drench to promote systemic uptake through the roots, ensuring uniform accumulation in plant tissues, which is more effective for strengthening plant defenses compared to localized foliar applications.

Each treatment group included three replicate pots. The silicon application started after plant establishment and continued for ten weeks. After the fifth application and upon reaching leaf maturity, representative leaf samples were harvested and analyzed for silicon accumulation. Leaf samples from each treatment were subsequently used as a substrate for mite infestation and observation of their biological parameters.

### 4.4. Silicon Accumulation Measurement

To quantify silicon content in the plant tissues, mature leaves were collected from each treatment group following the fifth week of application.

#### 4.4.1. Sample Preparation

Leaf samples were air-dried and then oven-dried at 60 °C overnight. The dried leaves were finely ground using a laboratory grinder, spread evenly on a clean plastic sheet, and homogenized. From this mixture, approximately 1 g of the ground material was sub-sampled by combining at least 10 small scoops using a spatula and stored in labeled plastic vials.

#### 4.4.2. Silicon Extraction

A 0.1 g sample of ground leaf tissue was placed into a 100 mL polypropylene tube. Then, 2 mL of 500 g/L hydrogen peroxide (H_2_O_2_) and 3 mL of 500 g/L sodium hydroxide (NaOH) were added. The mixture was stirred with a magnetic stirrer and incubated in an 85 °C water bath for 1 h to initiate digestion. Tubes were then sealed with stoppers and autoclaved at 123 °C and 1.5 bar pressure for 1 h. If undigested residues remained, an additional 1 mL of H_2_O_2_ was added, followed by another round of autoclaving. After digestion, 45 mL of deionized water (DI) was added, and the extracts were allowed to settle [23].

#### 4.4.3. Measurement of Silicon

From the settled extract, 1 mL of the supernatant was diluted with 19 mL of DI water (or 2 mL extract with 18 mL DI water for low Si content). A series of Si standards (0, 2, 4, 6, and 8 mL from a 50 ppm Si stock solution) were prepared with Merck Titrisol^®®^ Silicon standard and diluted to 50 mL in volumetric flasks. Each standard and sample received 1 mL of 500 g/L HCl and 2 mL of ammonium heptamolybdate solution. After 5–10 min, 2 mL of oxalic acid solution was added. Absorbance was read at 410 nm using a UV–visible spectrophotometer [23]. A calibration curve was plotted, and the Si concentration in unknown samples was calculated as follows:

$$Si\left(ppm\right)=ppm\;Si\left(from\;calibration\;curve\right)\times \frac{V}{Wt}\times Dilution\;Factor$$

where

*V* = Total extract volume (mL)

*Wt* = Dry weight of the sample (g).

### 4.5. Mite Rearing Protocol

Colonies of *T. macfarlanei* were maintained under controlled laboratory conditions (25 ± 1 °C, 65 ± 5% RH). The mites were reared on the undersides of clean, untreated *Lablab purpureus* leaves (approximately 25 cm^2^), placed on water-saturated polyurethane mats in 90 mm diameter Petri dishes. Leaf edges were wrapped in moistened tissue to prevent mite escape and maintain leaf turgor. Leaves were replaced regularly once feeding damage became extensive.

### 4.6. Data Collection on Biology and Reproduction

To examine the biological and reproductive responses of *T. macfarlanei* under varying silicon treatments, newly emerged females were selected at the teleiochrysalis (C3) stage for controlled mating. Each female was paired with a single male on a fresh bean leaf disc (approximately 2.5 cm in diameter) and allowed to mate. After successful copulation, the female was left on the leaf disc for further observation. The discs were maintained on water-saturated polyurethane foam inside plastic Petri dishes under controlled laboratory conditions (25 ± 1 °C, 65 ± 5% relative humidity). The edges of each leaf disc were wrapped in moist tissue paper to prevent mite escape and maintain leaf turgor [22].

Observations were conducted at 24 h intervals using a stereomicroscope. Data were recorded on several key parameters of female biology and reproduction. The pre-oviposition period was defined as the duration from the time of adult emergence to the deposition of the first egg. The oviposition period referred to the total number of days the female remained reproductively active and continued to lay eggs. The post-oviposition period was recorded from the time of the last egg laid until the female’s death. Fecundity was assessed by counting both the daily egg output and the total number of eggs produced by each female throughout her reproductive lifespan. Additionally, female and male longevities were recorded as the total number of days each individual lived from adult emergence until death [24].

These data were used to construct life tables and to quantify the effects of silicon treatment on the survival and reproductive fitness of *T. macfarlanei*.

### 4.7. Life Table Parameters

Developmental and reproductive data were analyzed using the age-stage, two-sex life table approach [25,26,27], which accounts for both sexes and variable developmental rates among individuals. The software TWOSEX-MSChart v 10.1.2024 [28] was used for calculations.

The following demographic parameters were estimated:

Age-stage-specific survival rate (*s_xj_*): Probability that a newly laid egg will survive to age *x* and stage *j*.

Age-specific survival rate (*l_x_*): Probability that a newly laid egg will survive to age *x*

$$l_{x}=\sum_{j=1}^{k}s_{xj}$$

Age-specific fecundity (*m_x_*): Average number of offspring produced per female at age *x*

$$m_{x}=\frac{\sum_{j=1}^{k}s_{xj}f_{xj}}{\sum_{j=1}^{k}s_{xj}}$$

Age-stage-specific fecundity (*f_xj_*): Number of eggs laid by individuals of age *x* and stage *j*.

Net reproductive rate (*R*_0_): The expected number of female offspring per individual during its lifetime.

$$R_{0}=\sum_{x=0}^{\infty }l_{x}m_{x}$$

Intrinsic rate of increase (*r*): Per capita growth rate of the population.

$$\sum_{x=0}^{\infty }e^{-r\left(x+1\right)}l_{x}m_{x}=1.$$

Finite rate of increase (*λ*): Population multiplication rate per unit time.

$$\lambda =e^{r}$$

Mean generation time (*T*): The average time from egg to the reproduction of offspring.

$$t=\frac{lnR_{0}}{r}\;.$$

Life expectancy (*e_xj_*): Expected remaining lifespan of an individual of age *x* and stage *j*.

$$e_{xj}=\sum_{i=x}^{\infty }\sum_{y=j}^{k}{S^{\prime }}_{iy}$$

Reproductive value (*v_xj_*): Future contribution of individuals of age *x* and stage *j* to the population.

$$v_{xj}=\frac{e^{r\left(x+1\right)}}{s_{xj}}\sum_{i=x}^{\infty }e^{-r\left(i+1\right)}\sum_{y=j}^{k}{S^{\prime }}_{iy}f_{iy}$$

### 4.8. Statistical Analysis

To estimate the variances and standard errors of all life table parameters, the bootstrap method was applied with 100,000 resampling iterations, ensuring robust statistical inference. This non-parametric approach minimizes variability in mean estimates and standard errors caused by random sampling. Comparisons among treatment groups (control, low Si, and high Si) were performed using paired bootstrap tests [29], with a significance level of *p* < 0.05 considered statistically significant. All computations were performed using the TWOSEX-MSChart program [28], specifically developed for life table analysis of arthropods with overlapping life stages and variable sex ratios.

## 5. Conclusions

This study clearly demonstrates that silicon (Si) has a significant suppressive effect on the developmental biology and reproductive potential of *Tetranychus macfarlanei* on country bean (*Lablab purpureus*). Silicon application, particularly at 56 ppm, markedly prolonged immature development, reduced female longevity and fecundity, shortened oviposition duration, and significantly lowered critical demographic parameters such as the intrinsic rate of increase (*r*), net reproductive rate (*R*_0_), and gross reproductive rate (*GRR*). These effects collectively indicate a strong inhibitory influence on population growth potential.

The findings suggest that Si induces both physical and physiological stress in mites, likely through reinforced plant tissues and enhanced biochemical defense responses. Moreover, the delayed generation time and diminished reproductive values observed under high Si treatment (56 ppm) emphasize the potential of silicon as an eco-friendly component in integrated mite management (IMM) strategies. The hormetic response observed at low silicon concentrations (28 ppm), where some parameters temporarily improved, also warrants further exploration into optimal dose selection.

Given the adverse impacts of synthetic acaricides on non-target organisms and the environment, silicon offers a promising alternative that aligns with sustainable agriculture principles. Future field validation across diverse agroecological settings and assessment of silicon’s compatibility with natural enemies and other IPM components will be essential to fully integrate this approach into pest management programs for *T. macfarlanei* and related species.

## Figures and Tables

**Table 1:** Application and accumulation of silicon (ppm, N = 5) in bean leaves used for *T. macfarlanei* life history analysis.

Treatment	Applied in Soil (ppm)	Measured in Leaf (ppm)
Control	0.00	116.60 ± 6.87
Low Silicon	28.00	161.20 ± 11.89
High Silicon	56.00	398.60 ± 9.42

**Table 2:** Developmental duration (days ± SE) of *T. macfarlanei* on silicon-treated bean plants at 25 °C and 65 ± 5% RH.

Treatment	Sex	N ^a^	Egg	Larva	Protonymph	Deutonymph	Egg-to-Adult
Control	♀	38	6.53 ± 0.14 a	2.53 ± 0.10 bc	2.26 ± 0.09 b	3.16 ± 0.09 abc	14.47 ± 0.19 bc
	♂	17	5.76 ± 0.22 bc	2.82 ± 0.20 a	2.41 ± 0.17 ab	2.88 ± 0.19 c	13.88 ± 0.37 c
Low Silicon	♀	30	6.30 ± 0.17 abc	2.97 ±0.15 ab	2.40 ± 0.09 ab	3.10 ± 0.14 ab	14.77 ± 0.23 ab
	♂	12	6.50 ± 0.19 a	2.83 ± 0.24 a	2.50 ± 0.19 ab	2.58 ± 0.26 cd	14.42 ± 0.31 abc
High Silicon	♀	34	5.94 ± 0.11 bc	2.88 ± 0.14 ab	2.59 ± 0.13 a	3.59 ± 0.21 a	15.00 ± 0.26 ab
	♂	16	6.19 ± 0.14 ab	3.00 ± 0.24 a	2.31 ± 0.12 ab	3.69 ± 0.30 a	15.19 ± 0.29 a

Values within columns followed by the same letter are not significantly different at *p* < 0.05 (paired bootstrap method). ^a^ Number of individuals tested.

**Table 3:** Reproductive traits and longevity of male and female *T. macfarlanei* on silicon-treated bean leaves.

Treatment	N ^a^	APOP ^b^	TPOP	Oviposition Days	Male Longevity	Female Longevity	Eggs per Female
Control	34	1.65 ± 0.13 a	16.03 ± 0.20 b	15.03 ± 1.54 ab	24.24 ± 2.83 a	32.68 ± 2.14 a	94.62 ± 10.44 a
Low Silicon	29	1.66 ± 0.13 a	16.41 ± 0.22 ab	19.07 ± 1.91 a	31.25 ± 4.25 a	37.77 ± 2.27 a	95.53 ± 11.45 a
High Silicon	34	1.68 ± 0.16 a	16.92 ± 0.28 a	10.88 ± 1.83 b	27.25 ± 2.13 a	26.26 ± 1.73 b	42.29 ± 9.12 b

APOP = adult pre-oviposition period, TPOP = total pre-oviposition period. ^a^ Number of individuals tested. ^b^ Mean values differ significantly at *p* < 0.05. Values followed by the same letters within a column are not significantly different at the 5% level using the paired bootstrap match method.

**Table 4:** Demographic parameters (mean ± SE) of *T. macfarlanei* under different silicon treatments.

Treatment	*R*_0_	*r*	*t*	*λ*	*GRR*
Control	53.62 ± 8.39 a	0.1679 ± 0.0061 a	23.71 ± 0.52 b	1.1829 ± 0.0072 a	126.30 ± 15.74 a
Low Silicon	47.77 ± 8.36 a	0.1541 ± 0.0074 a	25.10 ± 0.43 a	1.1666 ± 0.0086 a	116.17 ± 15.40 a
High Silicon	23.97 ± 5.76 b	0.1281 ± 0.0093 b	24.81 ± 0.60 ab	1.1366 ± 0.0106 b	92.66 ± 19.60 a

Net reproductive rate (*R*_0_), intrinsic rate of increase (*r*, day^−1^), mean generation time (*t*, day), finite rate of increase (*λ*), and gross reproduction rate (*GRR*). Mean value differs significantly at *p* < 0.05. Values followed by the same letters within a column are not significantly different at the 5% level using the paired bootstrap match method.

## Data Availability

The datasets generated in this study are available from the corresponding author on reasonable request.
